# Gynæcology

**Published:** 1921-06

**Authors:** R. S. S. Statham


### GYNÆCOLOGY.

**Incurable Carcinoma of the Cervix Uteri** occurs so frequently in middle-aged and elderly women, and causes so much distress not only to the patients but also to others, that it is not surprising that an immense amount of thought and experiment has been devoted to its alleviation. The combination of a large ulcerated surface with a copious discharge of a most offensive nature renders some form of local treatment essential, but local applications, such as iodoform, are practically useless. It is therefore in operative methods, and especially in electro-therapeutics, that recent advances have been made.

Total removal of the uterus and upper third of the vagina by Wertheim's method is now not so freely performed as a palliative operation, although it was at one time much advocated. The local recurrences were few, but the high primary mortality, averaging about 18 per cent. of all cases curable and incurable, has caused this operation to be confined mainly to cases in which there is a fair chance of cure. In dealing with this type of case any operation which is likely to effect a permanent cure is quite justifiable, but when the patient is inevitably doomed a treatment which will give the maximum of comfort with the minimum of risk is to be preferred.

Local removal with a sharp curette and treatment of the stump or shell of cervix and uterus with zinc chloride or 10 per cent. formalin is still the usual procedure. It is very efficacious for a time. The patient rapidly recovers from the slight disturbances of the operation, and local symptoms usually do not reappear for several months. The more fortunate patients die from secondary deposits or uræmic conditions before the pain, sloughing and fœtor re-commence, but the majority die a lingering death with much pain and discomfort.

*Diathermy* has given good results in the treatment of cervical carcinoma. The vaginal vault and cervix are ideal situations for its application, and the benefits are great and lasting. The local growth is removed as far as possible with a sharp spoon or curette, and the whole surface thus exposed is treated with a broad diathermy applicator until bubbles appear. No portion of the raw surface should be allowed to escape.

Great care is necessary to avoid involving the bladder or rectum in the area affected by the cauterisation, or a fistula connecting the vagina with one or other of these organs will result and the patient's condition be rendered more miserable.

The slough should separate between the seventh and eleventh days, and come away cleanly without any foul discharge. It is at this stage that the real danger of the treatment becomes manifest. Secondary hemorrhage of the most alarming severity often occurs. This can usually be controlled by firm packing, but in cases where the uterine artery has been involved in the slough hemorrhage has proved fatal. Two such cases have been reported by Corscaden.[1] Involvement of the ureter in the slough has been reported on one occasion. Apart from these accidents the results are excellent. Pain disappears at once, the raw area commences to scar over and contract, the discharge and fœtor practically cease, and marked improvement takes place in the general condition of the patient. Local recurrence is long delayed, giving welcome respite to the patient.

*Radium treatment* has also been employed in inoperable carcinoma of the cervix. Betrin[2] reports good results at the Geneva Clinic using 60 mgs. in lead filters 2 mm. thick. Chapuis[3] uses 150 mgs., placing it both inside the cervix and in the vaginal vault. Both these observers consider asepsis of the vagina essential, and both deprecate preliminary curettage. They insist upon the necessity of adequate filtration by lead or brass capsules 1.5 mm. thick enclosed in rubber envelopes. Protection of the rectum is essential, or troublesome diarrhœa supervenes.

*Percy's Cold Cautery* is recommended as an ideal method of treatment, but requires an elaborate technique. Percival Cole[4] has recently reported a series of 43 cases with the following results: an average duration of 16 months with complete relief of symptoms in all cases save 8, which died within 3½ months; four cases lived for 2 years and one for 3 years and 2 months. The operation is performed in two stages.

(*a*) Intra-abdominal. All the intestines are pushed into the upper abdomen after freeing any adhesions, and are packed away from the pelvic cavity with hot cloths. The internal iliac and ovarian vessels are then tied on both sides.

(*b*) The vaginal operation. This is immediately proceeded with through a special water-cooled speculum, and the cautery is guided into position by the assistant's hand in the abdominal cavity. The whole interior of the uterus, the cervix and the bases of the broad ligament are then " cooked " till all the carcinomatous area has been attacked. The abdomen is then closed, the operation lasting about 1½ hours. Secondary hemorrhage is rare, and if it occurs is slight in amount. Vesico-vaginal fistula is the commonest complication, 7 cases in 43. Two fatal cases are reported from the Johns Hopkins Hospital[5] and one by Bancroft.[6] The operation mortality compares favourably with any other method of treatment, while the patient's comfort and expectation of life are greatly improved. Cole[4] advocates the use of radium in conjunction with this method, and points out that it should not be used in local recurrences after panhysterectomy owing to the proximity of the bladder.

REFERENCES.

1 *Med. Rec.*, 1918, xciii. 745.
2 *Gynécologie*, Aug., 1920 ; abstr. in *Brit. M. J.*, 1921, I. Epit., p. 43.
3 Quoted by Betrin, *loc. cit.*
4 *Lancet*, 1921, i. 166.
5 *Johns Hopkins Hosp. Rep.*, 1919, xviii. 305.
6 *Med. Rec.*, 1916, lxxxix. 714.

R. S. S. STATHAM.

